# Stable isotope ratios of nitrogen and carbon as biomarkers of a vegan diet

**DOI:** 10.1007/s00394-022-02992-y

**Published:** 2022-09-10

**Authors:** Jutta Dierkes, Stefan Dietrich, Klaus Abraham, Bernhard H. Monien, Adrian McCann, Katrine Borgå, Cornelia Weikert

**Affiliations:** 1 Department of Clinical Medicine, Centre for Nutrition, Mohn Nutrition Research Laboratory, University of Bergen, Haukelandsbakken 15, 5021 Bergen, Norway; 2 Department of Medical Biochemistry and Pharmacology, Haukeland University Hospital, Bergen, Norway; 3 Department of Food Safety, German Federal Institute for Risk Assessment, 10589 Berlin, Germany; 4 Bevital AS, Bergen, Norway; 5 Department of Biosciences, University of Oslo, Oslo, Norway

**Keywords:** δ15N, δ13C, Stable isotope ratios, Vegan diet, Biomarkers, Dietary intake data., Nitrogen isotopes

## Abstract

**Purpose:**

Dietary biomarkers can potentially overcome the limitations of self-reported dietary data. While in ecology and archaeology, stable isotope ratios of carbon and nitrogen are widely used as biomarkers, this is not the case in nutrition research. Since the abundance of the 13C and the 15N isotope differ in food sources from plant and animal origin, stable isotope ratios of carbon and nitrogen (δ13C and δ15N) may differ in human biological material. Here, we investigated the stable isotope ratios of nitrogen and carbon in serum and urine from vegans and omnivores.

**Method:**

Measurement of δ15N and δ13C in serum and 24 h urine was performed by Elemental Analyzer–Isotope Ratio Mass Spectrometer in the cross-sectional study “Risks and Benefits of a Vegan Diet”. The study included 36 vegans and 36 omnivores with a median age of 37.5 years (matched for age and sex), who adhered to their diet for at least 1 year.

**Results:**

Both δ15N and δ13C were significantly lower in both the serum and 24 h urine of vegans compared to omnivores. δ15N either in serum or urine had 100% specificity and sensitivity to discriminate between vegans and omnivores. Specificity of δ13C was also > 90%, while sensitivity was 93% in serum and 77% in urine.

**Conclusion:**

δ15N both in serum and urine was able to accurately identify vegans and thus appears to be a promising marker for dietary habits.

**Supplementary Information:**

The online version contains supplementary material available at 10.1007/s00394-022-02992-y.

## Introduction

Dietary intake is widely recognized as one of the most important lifestyle factors that influence both human health and planetary health. Meat intake in particular has been linked to non-communicable diseases, and its production has implications for land and water use, as well as greenhouse gas production [1]. Diets that exclude meat, or more radically all animal products, have attracted increasing attention in the Western world.

Even though the importance of diet in relation to health outcomes has been identified, challenges concerning the validity and reliability of dietary intake data continue to undermine research in this field. Methods for assessing dietary intake typically involve self-report and rely on memory recall and objectivity. Dietary intake methods based on self-reported data are prone to be influenced by factors such as the social desirability of foods, lack of memory or lack of consciousness that a food item(s) have been consumed, and lack of ability to estimate portion sizes or amounts of foods consumed [2]. Thus, there is a need for a more objective assessment of dietary intake, and enormous progress has been made during the last decade concerning the use of food-specific biomarkers [3, 4].

Stable isotope ratios are among the biomarkers that have been investigated as indicators of meat and fish [5], and added sugar intake [6]. Stable isotopes are atoms of the same element that differ in the number of neutrons in the nucleus and thus differ in their atomic mass. In nature, each element occurs as a mixture of its isotopic forms, but metabolic rates in plants and animals are usually different for certain isotopes, resulting in small differences in the permille range. The isotope distribution in samples is usually expressed relative to the distribution of universal standard material, which is usually limestone (V-PDB) for carbon and nitrogen in air for nitrogen [7]. The ratio of naturally occurring stable isotopes of carbon (13C/12C ratio expressed as δ13C, sometimes also called CIR) and nitrogen (15N/14N) ratio expressed as δ15N, also called NIR) have been used extensively in archaeological and ecological studies, and their use in dietary assessment studies is increasing [5, 7–10].

In biology, stable isotopes have been also used to characterize trophic positions in the food web. Due to greater retention of the heavier 15N isotope than the lighter 14 N isotope in the production of nitrogenous waste, the nitrogen ratio of 15N to 14N (δ15N) shows a stepwise enrichment from food producers to food consumers and is therefore indicative of relative trophic position [7, 11, 12]. Thus, δ15N can differentiate between trophic levels, as the relative abundance of the heavy nitrogen isotope, increases by approximately 2–4‰ per increasing trophic levels in the food web [7]. Although the trophic level or position is a well-known concept within biology, it has not been used in the field of human nutrition science. Based on the trophic model, humans who consume omnivorous diets would accordingly be seen as ‘higher predators’, while vegans would be on a lower trophic level as they only consume plant-based food. Vegetarians, who do not consume meat, but milk and dairy, eggs and honey, would be between vegans and omnivores.

While the δ15N ratio in a food web reflects the trophic position, differences in the δ13C are more dependent on the type of plants consumed. C3 plants (the majority of food plants such as wheat, rice, or beans) to a greater degree than C4 plants utilize the ^12^C rather than ^13^C in the photosynthesis when trapping/converting C from CO_2_ reflected by a lower δ13C value. C4 plants, among them sugar cane, corn and sorghum, have δ13C values approximately 12–13‰ higher than C3 plants. This difference can be used to measure the consumption of added sugar made from either sugar cane or corn while added sugar produced from sugar beet (C3 plant) would not show any difference in the δ13C [6, 13, 14]. However, carbon atoms in the diet are derived from all macronutrients and are thus more difficult to interpret. Indeed, the feed of husbandry animals like pork is mainly based on corn, which would reflect the δ13C of C4 plants. Indeed, δ13C has recently been suggested as a marker for animal protein intake [15].

Stable isotope ratios as dietary biomarkers can be measured in different tissues or body fluids, including skin [16], urine [5, 17], fingernails [18], exhaled air [13, 19], hair [8, 20], blood [6] and serum [5, 21]. Stable isotope ratios in these biological specimens may reflect different time periods and varying nutrient turnover rates [7].

Here, we investigate in an exploratory manner whether the stable isotope ratios of δ13C and δ15N in serum and 24 h urine can distinguish between healthy vegans and omnivores in a cross-sectional study. In addition, we investigate the discriminative power of δ13C and δ15N in comparison to two other dietary biomarkers for dairy and meat intake, plasma pentadecanoic acid (15:0) and plasma 1-methylhistidine [3].

## Subjects and methods

This is a cross-sectional study investigating the nutritional status of vegans, compared to omnivores. The recruitment process for healthy volunteers, aged 30–60 years, is described elsewhere [22]. Briefly, the 36 vegans and 36 omnivores of the study “Risks and Benefits of a Vegan Diet” (RBVD) were recruited in Berlin (Germany) at the German Federal Institute for Risk Assessment (BfR) in the period from January to July 2017, matched for age and sex. The sample size for this study is based on the power calculation for the primary research question (bone health in vegans compared to omnivores) and due to the exploratory nature of the current analysis, a sample size calculation is not provided here [23]. The study was approved by the Ethics Committee of Charité—Universitätsmedizin Berlin (no. EA4/121/16). A flow chart of the study process is shown in Supplemental Fig. 1. Written informed consent was obtained from all participants during the first visit.

**Fig. 1:**
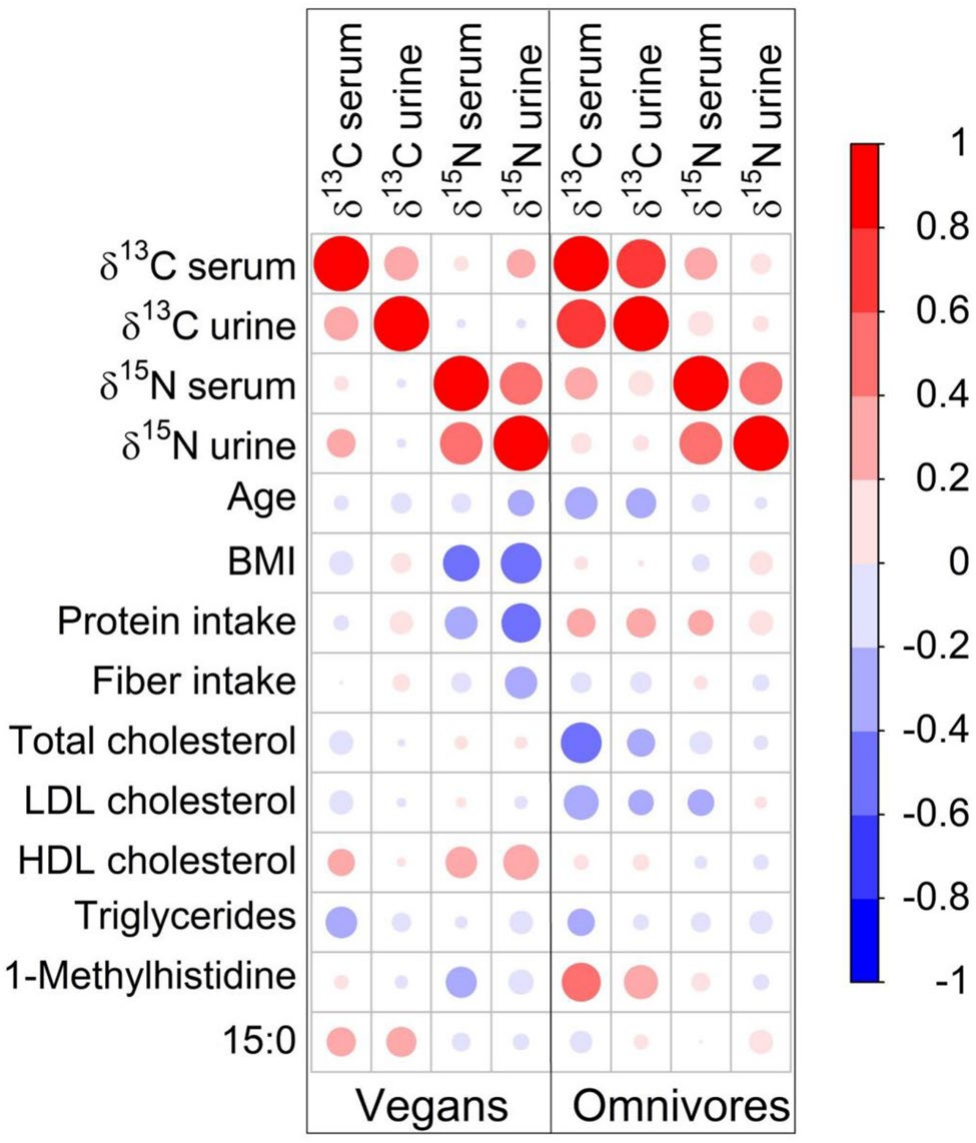
Spearman correlation matrix for stable isotope ratios (δ15N, δ13C) in urine and serum with age, BMI, dietary intake and serum or plasma variables (lipids, 15:0 and 1-methylhistidine) in vegans (*n = *36) and omnivores (*n = *36). δ15N nitrogen stable isotope ratio, δ13C carbon stable isotope ratio, 15:0 pentadecanoic acid;

As an inclusion criterion, vegans should follow the vegan diet for at least a year, and omnivores should consume at least three times meat or two times meat and two times processed meat per week. Dietary intakes were recorded using three-day weighed food protocols. With the help of the German Nutrient Database (BLS, Bundeslebensmittelschlüssel) Version 3.02, the mean daily intake of food items, macronutrients and micronutrients was calculated. Information about age, educational attainment, and lifestyle factors were collected using tablet-based questionnaires. Height and body weight, waist circumference, and blood pressure were measured using standardized methods.

From all participants in the study, 60 mL of blood was obtained. Blood lipids and creatinine were measured in a certified routine laboratory (Labor 28 GmbH, Berlin, Germany) by standard methods on the same day of blood collection. 24 h urine was collected by the participants, and urine creatinine concentrations were determined also determined on the day of the visit to the study center. All other biochemical analyses were performed on samples stored at a temperature of − 80 °C. Urine was collected on the days prior to the visit to the study center and were done from Sunday to Thursday.

### Stable isotope ratio and biomarker assessment

Stable isotope ratios δ13C and δ15N were measured at the Stable Isotope Laboratory at the University of Oslo (UIO:CLIPT), using a method described by Kraft [24]. Briefly, serum (8 µL) and urine (15µL) were pipetted into tin capsules and air dried. The δ15N and δ13C were measured simultaneously using an Elemental Analyzer (EA) IsoLink Isotope Ratio Mass Spectrometer (IRMS) System, consisting of a Flash EA and a DeltaV IRMS (Thermo Scientific, Germany). The δ13C and δ15N values were normalized to the Vienna Pee Dee Belemnite (VPDB) and AIR scales, respectively, using two different internal reference materials incorporated into each analytical run: JGLUT (L-glutamic acid; δ13C = − 13.43 ‰; δ15N − 4.34‰) and POPPGLY (glycine; δ13C = − 36.58 ‰; δ15N 11.25 ‰) (both from Fisher Scientific). An additional quality control material, JALA (alanine, calibrated value δ13C = − 20.62 ‰; measured value (*n = *40) − 20.58 ± 0.07 ‰; calibrated value: δ15N − 3.16 ‰, measured value (*n = *31) − 3.23 ± 0.06 ‰)(Fisher Scientific) was incorporated into every run. δ13C of both reference materials and quality control sample were calibrated to the VPDB scale using LSVEC (lithium carbonate, δ13C = − 46.6 ‰) and NBS-19 (calcium carbonate, δ13C = 1.95‰) (both obtained from the International Atomic Energy Agency, Austria). The δ15N values were calibrated to the AIR scale using USGS40 (L-glutamic acid, δ15N − 47.57 ‰) and USGS41 (L-glutamic acid, δ15*N = *47.57‰) (both obtained from the United States Geological Survey). Analytical precision was based on repeated analyses of quality assurance material JALA (Fisher Scientific).

The fatty acid pentadecanoic acid (15:0) was measured as % of all fatty acids in plasma phospholipids at the German Institute of Human Nutrition Potsdam-Rehbrücke (Germany). The method for 15:0 measurement was described previously by Weitkunat [25]. In addition, we measured 1-methylhistidine (m1His) in plasma at Bevital AS (Bergen, Norway, http://www.bevital.no). 1-methylhistidine was quantified using an isotope-labeled internal standard to an existing assay utilizing liquid chromatography combined with tandem mass spectrometry, as previously described [26].

### Statistics

The study was powered by a primary research question about differences in bone health between vegans and omnivores. Data were analysed exploratory to answer the research questions.

Variables were reported using mean and standard deviation (SD) for normally distributed variables, median and interquartile range (IQR) for non-normally distributed variables, and relative percentages for categorical variables. Differences between vegans and omnivores were tested using a Chi-Square test for categorical variables and a Student’s *T*-test (normally distributed) or Kruskal–Wallis test (non-normally distributed) for continuous variables. Normal distribution of variables was prooven using the Shapiro–Wilk test, which indicated the non-normal distribution of δ13C, δ15N, 15:0 and 1-methylhistidine. Spearman correlations were calculated to investigate potential correlations between isotopes and variables of interest. To study the discrimination performance of biomarkers (δ13C, δ15N, 15:0 and 1-methylhistidine) regarding the dietary group (vegan vs. omnivorous diet), receiver operating characteristic curves (ROC) were plotted using the R package ROCit with a parametric binormal approach. The ROC curve represents a plot of sensitivity versus false-positive rate (1-specificity) of logistic regression prediction models (Diet ~ Exposure). The area under the ROC curve (AUC) represents the probability that the prediction model assigns a true vegan as vegan compared to an omnivore. The AUC may range from 0.5 indicating no discrimination to 1.0 indicating perfect discrimination. Scatter plots were used to derive cut-offs for the discrimination analysis (sensitivity and specificity).

For statistical analyses of data, SAS software (version 9.4, SAS institute, Cary, N.C., USA) and R software (version 3.6.3) was used. Even though the analyses in this report are exploratory, a p value of 0.05 was regarded as significant [27].

## Results

### Cohort characteristics

In total, 72 healthy volunteers, 36 vegans and 36 omnivores (each 50% men) were included. Median age was 37.5 years (min–max: 30 –57), and median duration of vegan diet was almost 5 years. Main characteristics of the study sample are presented in Table 1.

**Table 1:** Characteristics of the study sample

	Vegans (*n = *36)	Omnivores (*n = *36)	*p* values
Men [n (%)]	18 (50)	18 (50)	1.00
Age (years)	37.5 (32.5–44.0)	38.5 (32.0–46.0)	0.75
Body weight (kg)	70.1 (± 13.9)	73.6 (± 10.3)	0.24
BMI	22.9 (± 3.2)	24.0 (± 2.1)	0.08
Creatinine serum (mg/dL)	0.82 (± 0.15)	0.89 (± 0.15)	0.046
Duration of vegan diet (years)	4.8 (3.1–8.7)	n.a	
Education [*n* (%)]			0.60
Lower	0 (0)	1 (2.8)	
Middle	11 (30.6)	11 (30.6)	
High	25 (69.5)	24 (66.7)	
Physical activity (h/week)	2.8 (0.9–3.8)	2.3 (1.2–4.1)	0.69
Smoking behavior [*n* (%)]			0.3
Never-smoker	24 (66.7)	21 (58.3)	
Former smoker	8 (22.2)	6 (16.7)	
Smoker	4 (11.1)	9 (25.0)	
Dietary intake			
Total energy (kcal)	2270 (1800–2762)	2386 (2081–2737)	0.32
Protein (g/d)	72 (55–92)	86 (71–107)	0.02
Fat (g/d)	86 (64–111)	104 (88–143)	0.004
Carbohydrates (g/d)	259 (212–371)	230 (199–291)	0.12
Fibre (g/d)	46 (34–58)	24 (19–30)	< 0.0001
Food intake (g/d)			
Poultry	0 (0–0)	15 (0–61)	
Red meat	0 (0–0)	18 (0–85)	
Processed meat	0 (0–0)	32 (3–77)	
Fish	0 (0–0)	0 (0–39)	
Dairy	0 (0–0)	351 (185–481)	
Egg	0 (0–0)	17 (0–40)	

Variables expressed as percentage (*n*), mean (± SD) or median (IQR). Differences between the diet groups were assessed using chi-square tests for categorical variables by Chi-Square test, Student’s *T*-test for normally distributed continuous variables and Kruskal–Wallis test for not normally distributed continuous variables. Education categories are low (no qualifications), middle (vocational training) or high (university or other higher education degree), smoking categories are never smokers, former smokers (smoking previously but stopped) and smoker (current smoker)

### Differences of δ15N and δ13C in serum and urine

Table 2 shows the measured δ13C and δ15N values in serum and 24 h urine samples in both vegans and omnivores. In both groups, stable isotope ratios for carbon and nitrogen, were lower in urine compared to serum. Compared to omnivores, vegans had lower levels of δ13C and δ15N in urine and serum, respectively. The δ15N in vegans was approximately 2 ‰ lower in urine and approximately 1.5 ‰ lower in serum than in omnivores. For δ13C, the difference between vegans and omnivores was approximately -1‰ in urine and -1.5‰ in serum.

**Table 2:** Dietary biomarkers in vegans and omnivores

	*N*	Median (IQR)	Min	Max	*p* value
δ15N urine					
Vegans	36	2.74 (2.51; 3.18)	1.76	3.99	
Omnivores	36	4.69 (4.39; 5.06)	4.04	5.67	< 0.0001
δ15N serum					
Vegans	36	7.68 (7.48; 7.87)	6.61	8.43	
Omnivores	36	9.49 (9.32; 9.65)	9.04	10.31	< 0.0001
δ13C urine					
Vegans	36	− 25.38 (− 25.76; − 25.20)	− 26.64	− 24.15	
Omnivores	36	− 24.34 (− 24.75; − 23.88)	− 25.56	− 21.06	< 0.0001
δ13C serum					
Vegans	36	− 24.35 (− 24.54; − 24.16)	− 24.88	− 23.05	
Omnivores	36	− 22.76 (− 23.08; − 22.4)	− 23.77	− 22.11	< 0.0001
15:0 (%)					
Vegans	36	0.15 (0.13; 0.16)	0.09	0.22	
Omnivores	36	0.26 (0.22; 0.30)	0.16	0.40	< 0.0001
1-methylhistidine (µmol/L)					
Vegans	36	0.38 (0.31; 0.46)	0.10	0.69	
Omnivores	36	3.57 (1.72; 10.25)	0.40	37.2	< 0.0001

Stable isotope ratios (‰) for carbon (δ13C) and nitrogen (δ15N) in serum and 24 h urine in and plasma concentrations of pentadecanoic acid (15:0) and 1-methylhistidine of vegans and omnivores.. The stable isotope ratios have been obtained against standard material (Vienna Pee Dee Belemnite (VPDB) for carbon and air for nitrogen, as described in Methods) which explains the negative values for δ13C. δ15N nitrogen stable isotope ratio, δ13C carbon stable isotope ratio

The biomarkers 15:0 (% of total fatty acids) and 1-methylhistidine in plasma were also highly significantly different between vegans and omnivores, with higher values in omnivores compared to vegans for both biomarkers.

### Correlations of biomarkers

There were no differences between men and women for either δ15N nor δ13C. In omnivores, there was a strong correlation of δ13C in serum with δ13C in urine (*r = *0.78), which was much weaker in vegans (*r = *0.36). Correlation of δ15N in serum with δ15N in urine was, however, similar in both omnivores (*r = *0.59) and vegans (*r = *0.58). The strength of the correlations of both δ15N and δ13C with other factors such as age, BMI, or lipids, differed in the omnivorous group compared with the vegan group (Fig. 1).

### Sensitivity and specificity analyses for biomarkers

**Fig. 2:**
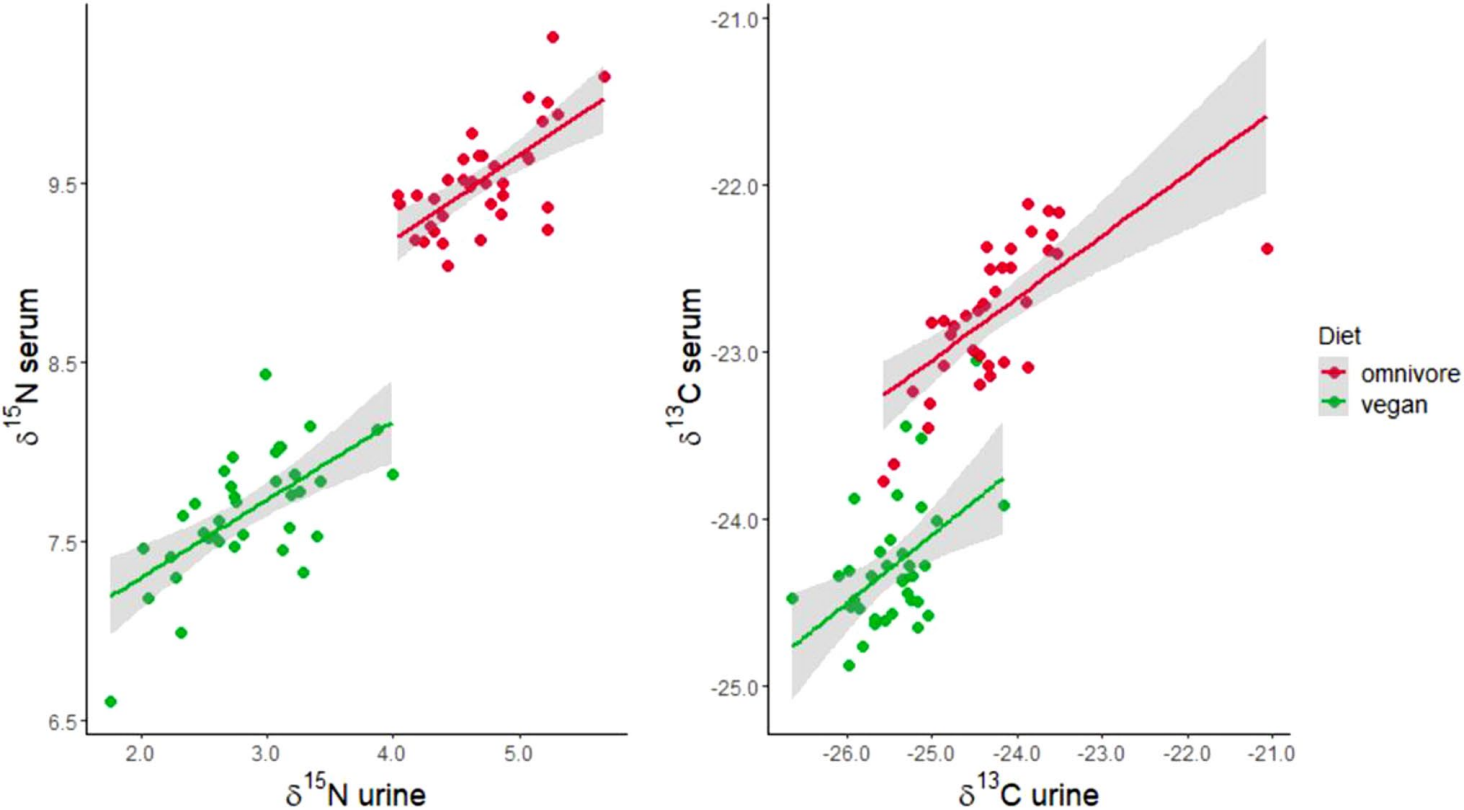
Scatter plots of δ15N and δ13C in urine and serum, separated for the two dietary groups

**Fig. 3:**
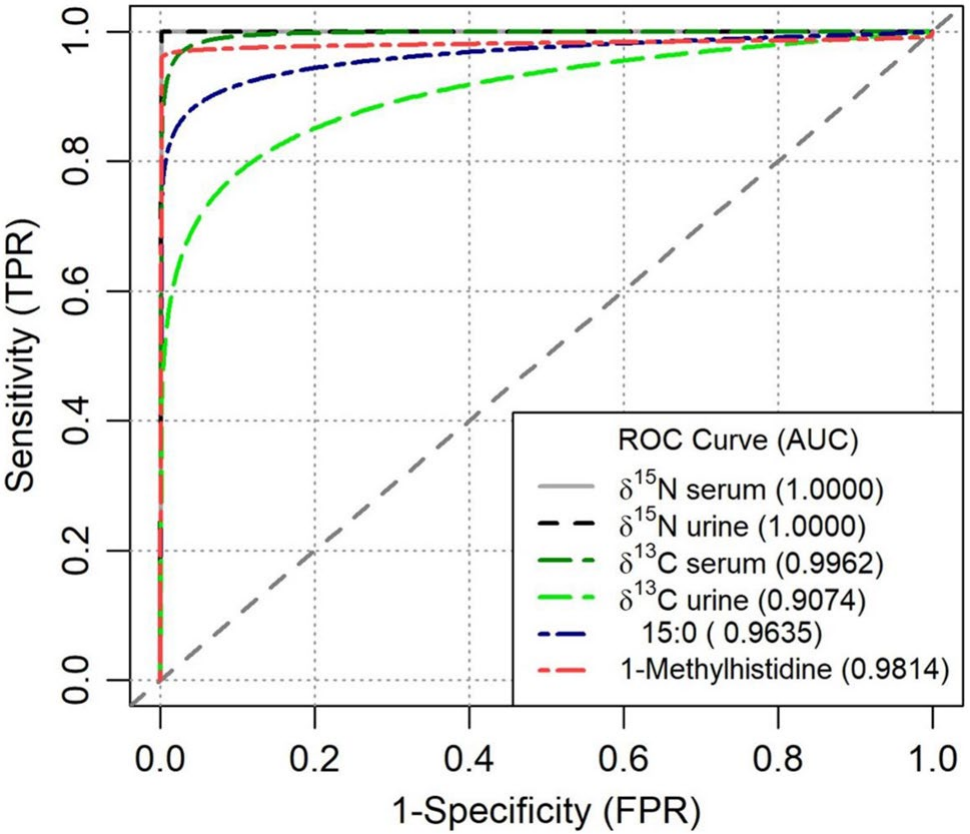
Receiver characteristic curve for δ15N, δ13C, 15:0, 1-methylhistidine to distinguish between vegans (*n = *36) and omnivores (*n = *36). (TP*R = *true positive rate, FP*R = *false positive rate)

The sensitivity and specificity of the SIR biomarkers, 15:0, and 1-methylhistidine to predict whether an individual practices a vegan or omnivorous diet was evaluated by ROC analyses. The δ15N in serum and urine had 100% sensitivity and specificity to discriminate between vegans and omnivores. δ13C, 15:0 and m1His also had high sensitivity and specificity. Results are shown in Figs. 2, and 3 and in Table 3.

**Table 3:** Estimated sensitivity and specificity of δ13C δ15N, 15:0 and 1-methylhistidine to distinguish between vegans and omnivores based on cut-offs

Parameter AND cut-offs	Vegan diet		Sensitivity	Specificity
	Yes	No		
δ13C serum (‰)				
< − 23.5	34	2	94%	94%
> − 23.5	2	34		
δ13C urine (‰)				
< − 24.5	34	12	94%	66%
> − 24.5	2	24		
δ15N serum (‰)				
< 8.5	36	0	100%	100%
> 8.5‰	0	36		
δ15N urine (‰)				
< 4.0	36	0	100%	100%
> 4.0	0	36		
15:0 (%)				
< 0.2	34	6	94%	83%
> 0.2	2	30		
1-methylhistidine (µmol/L)				
< 1	36	8	100%	78%
> 1	0	28		

## Discussion

This cross-sectional study investigated biomarkers of vegan or omnivorous diet in plasma and 24 h urine. The main results are that δ15N and δ13C from vegans are much lower both in plasma and in 24 h urine, compared to omnivores with at least three meat consumption occasions per week. In particular, δ15N seems to be well suited to discriminate between participants following a vegan or an omnivorous diet. Further, in a ROC analysis, δ15N performed better than 1-methylhistidine or 15:0, which are discussed as specific biomarkers of meat or dairy intake, respectively.

Stable isotope ratios have been used for several years to characterize dietary habits in contemporary humans [8, 9], including both vegan or vegetarian diets and different body tissues or fluids including hair [8, 9, 21], fingernails [18]**,** whole blood [5], or serum [28].

Most of these studies reported lower δ15N in vegans compared to omnivores, even though the sample size in most studies was low and not exceeding 16 persons, and the time period since these persons followed a vegan diet was only occasionally provided [8, 9, 18, 21]. In our study, adherence to a vegan diet for at least 1 year was an inclusion criterion. Further, diets in the omnivore groups are often not characterized in detail [8, 18, 25, 28], unlike our omnivorous participants who reported consumption of meat or meat products at least three times per week [22]. Most studies did not report differences between vegans and omnivores for δ13C (9, 18, 21). Three studies compared also stable isotope ratios of vegans and vegetarians and reported either no differences between these (21, 28) or lower ratios in vegans for δ15N only (8).

Different tissues have been used in nutritional stable isotope ratio research, including hair [9, 21, 29, 30] and urine [5, 17], which can both be obtained non-invasively. Indeed, stable isotope ratios in different tissues may be different. Nash et al. measured both δ15N and δ13C in plasma, red-blood cells and hair, and reported lower δ15N in erythrocytes compared to plasma and hair, while δ13C was higher in hair compared to plasma and RBC [29]. Very similar findings were reported for hair, plasma and erythrocytes [30]. Kuhnle reported lower δ15N values in urine compared to whole blood for δ15N, while differences for δ13C were less evident [5]. Hülsemann reported variation in the δ15N of urinary urea in 69 samples obtained from 8 omnivorous participants over a period of 48 to 104 h, the mean δ15N was 4.4 ± 0.6‰ [17]. We are not aware of studies that compared spot urine and 24 h urine, and how far short-term changes in hydration and diuresis affect SIRs in urine.

Generally, it is understood that the overall δ15N and δ13C in body fluids and tissues should reflect the stable isotope ratios observed in food sources. However, there are not many contemporary analyses of stable isotope ratios in foods. According to Hülsemann, cereals and legumes have lower δ15N values compared to meat and dairy (examples from their analyses: whole grain bread 2.3‰, soya Bolognese 2.4‰, yoghurt 6.0‰, beef roulade 7.4‰) [31].

Thus, lower δ15N in vegans may reflect the consumption of plant-based protein sources instead of animal protein with higher δ15N. We observed lower urinary δ15N than plasma δ15N (both in vegans and omnivores) which may reflect to a stronger extent the preferred excretion of 14 N [17]. While 14 N is preferably excreted in urine, 15 N is retained and leads to higher δ15N in serum. There are also some other noteworthy dietary differences between vegans and omnivores. Vegans reported lower total protein intake, although on average, it still exceeded 1 g per kg body weight and lower fat and higher carbohydrate intake (Table 1). The lower protein intake may also lead to differences in urinary nitrogen excretion, however, as we did not measure urinary nitrogen, this remains speculative.

In comparison to the δ15N ratio, specificity, and sensitivity of the δ13C ratio to distinguish between vegans and omnivores were lower, but comparable to two other biomarkers of animal food intake. The fatty acid, 15:0, has been suggested as a biomarker of dairy intake and has shown a good correlation with reported dairy intakes in epidemiologic studies [32], although it has not been widely used. Recently it has been shown that odd-chain fatty acids (15:0 and 17:0) can also be synthesized internally from propionic acid, derived from dietary fiber, which would limit their use as a biomarker of dairy intake, although this seemed to be more an issue for 17:0 [25]. 1-methylhistidine has recently been proposed as a biomarker of cod and salmon intake in a randomized controlled trial [33] and as a biomarker of animal protein intake in clinical [34] and epidemiologic studies [35]. Significant differences in 1-methylhistidine urinary concentrations has also been described in vegans and non-vegans in the Adventist Health Study 2 [36]. Indeed, highly significant differences were observed for both markers upon a comparison of the two groups in the present study. Yet, both markers showed lower sensitivity and specificity than δ15N. To our knowledge, the present study is the first to have measured and evaluated these biomarkers in combination.

Although the cross-sectional nature and sample size of our study precludes more advanced statistical analyses, the findings are nonetheless promising and should inform future work investigating reliable biomarkers of dietary intake and patterns. The results warrant therefore confirmation in studies with a more advanced study design. Of note, we relied on self-reported dietary habits when grouping the participants into a vegan or omnivorous diet. However, the risk of misclassification in this study seems to be low as participants filled in 3 days of dietary records and the call for participation was specifically addressing vegan diet and omnivorous diet, including the duration of a vegan diet and the requirement of 3 or more meat consumption occasions per week.

In conclusion, the RBVD study included strict definitions of a vegan and omnivorous diet, implemented different nutritional status measurements, and provided the opportunity for stable isotope ratios and dietary biomarkers such as 15:0 percentage or 1-methylhistidine concentration to be investigated together for the first time. Using these biomarkers in combination may be promising and help to master the challenge to distinguish between for instance vegetarian and flexitarian diets, even if the absolute differences in stable isotopes between vegans and omnivores were small. Further research should focus on the added value of these combinations of biomarkers to monitor dietary changes, and whether stable isotope ratios alone or in combination with other biomarkers provide greater sensitivity, specificity, and ultimately reliability and reproducibility when distinguishing between omnivores, vegetarians, and vegans.

## Supplementary Information

Below is the link to the electronic supplementary material.

Supplementary file1 (JPG 95 KB)
