# Supplementary figures and images for: Stable isotope ratios of nitrogen and carbon as biomarkers of a vegan diet
Source: Eur J Nutr. 2022 Sep 10;62(1):433–41. doi: 10.1007/s00394-022-02992-y (PMC9899720; doi:10.1007/s00394-022-02992-y)

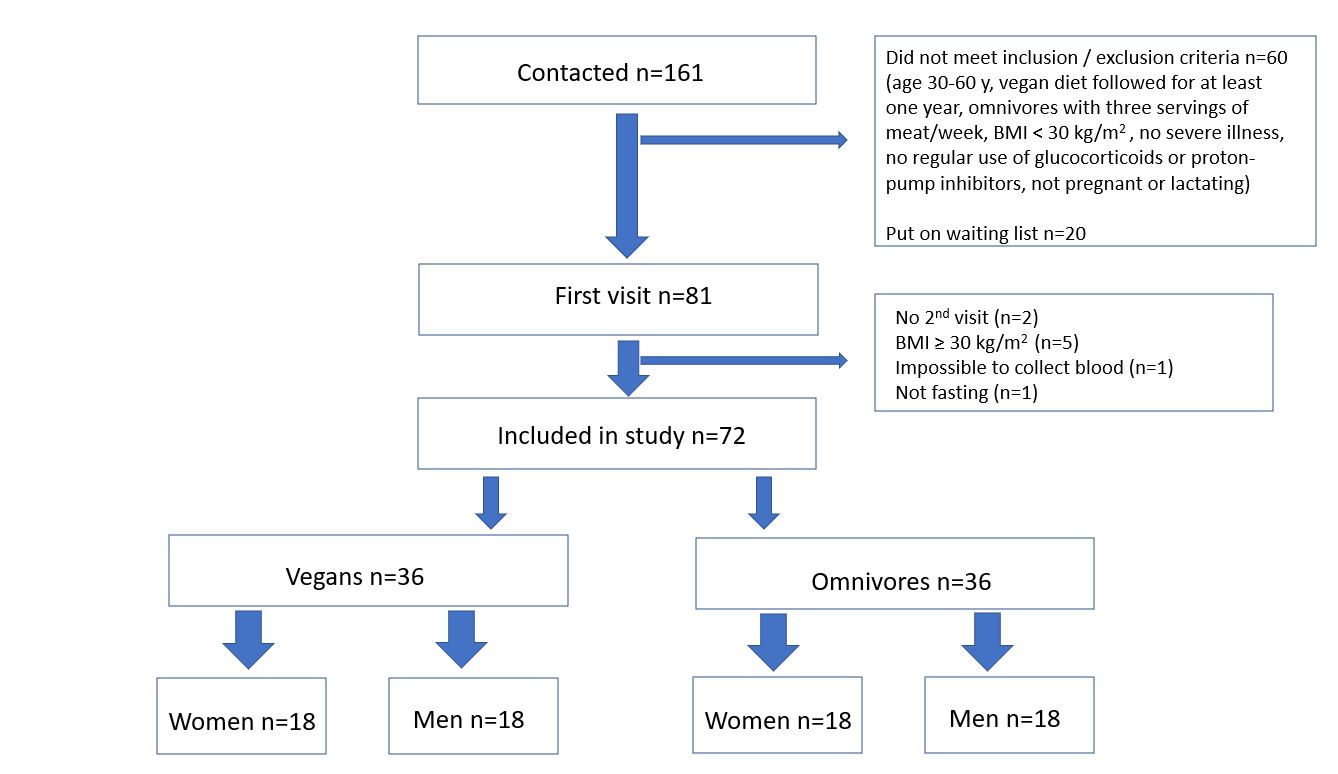

Supplement: Supplementary file 1 — Supplementary file1 (JPG 95 KB) [file 394_2022_2992_MOESM1_ESM.jpg]
